# Case Report: Transient severe T cell lymphopenia in a patient with Cornelia de Lange Syndrome captured by TREC screening

**DOI:** 10.3389/fimmu.2026.1729053

**Published:** 2026-03-03

**Authors:** Hope Retif, Devyn Rolhfs Rivera, Jariya Upadia, Christian Wysocki, Andrew Abreo, Luke A. Wall

**Affiliations:** 1 Department of Pediatrics, Louisiana State University Health Sciences Center, New Orleans, LA, United States; 2 Manning Family Children’s, New Orleans, LA, United States; 3 Division of Allergy and Immunology, Departments of Pediatrics and Medicine, Ochsner Medical Center, New Orleans, LA, United States; 4 Hayward Genetics Center, Department of Pediatrics, Tulane University School of Medicine, New Orleans, LA, United States; 5 Division of Allergy and Immunology, Departments of Pediatrics and Medicine, University of Texas Southwestern Medical Center, Dallas, TX, United States; 6 Division of Allergy and Immunology, Department of Pediatrics, Louisiana State University Health Sciences Center, New Orleans, LA, United States

**Keywords:** Cornelia de Lange Syndrome (CdLS), newborn screening, nipped B-like (NIPBL), T cell lymphopenia, T-cell receptor excision circle (TREC)

## Abstract

Cornelia de Lange Syndrome (CdLS) is a rare multisystem disorder characterized by craniofacial dysmorphism, growth restriction, limb anomalies, intellectual disability, and mild to moderate immune abnormalities. We present the case of a newborn female with CdLS who was found to have severe transient T-cell lymphopenia following abnormal newborn T-cell receptor excision circle (TREC) screening. She was treated with immunoglobulin replacement and antimicrobial prophylaxis. She experienced normalization of T cells and no severe infections over a two-year period. To our knowledge, this is the first detailed report of a case of CdLS presenting with profound T-cell lymphopenia identified by newborn screening, underscoring the utility of TREC screening in syndromic infants and the need for further study of immune defects in CdLS.

## Introduction

Cornelia de Lange Syndrome (CdLS) is a rare disorder characterized by a spectrum of clinical features including prenatal onset growth restriction, upper-limb reduction defects, distinctive craniofacial abnormalities, hypertrichosis, and intellectual disability. Its estimated prevalence is as high as 1 in 10,000 (1). Affected individuals are typically recognized by distinctive craniofacial features including synophrys, high-arched eyebrows, long eyelashes, a short nasal bridge with anteverted nares, small widely spaced teeth, and microcephaly. Over time, most are found to have intellectual disability, with an average intellectual quotient (IQ) of approximately 53 (2). Additional associated findings include cardiac septal defects, gastrointestinal dysfunction, hearing loss, myopia, cryptorchidism or hypoplastic genitalia, as well as autistic or self-injurious behaviors (3). Infections — such as pneumonia, viral respiratory infections, and sepsis — have been described as significant causes of morbidity and mortality in these patients (1). Previously described immunologic findings in CdLS include mild B cell lymphopenia, reduced switched memory B cell numbers, decreased serum immunoglobulin levels, and qualitative T cell abnormalities (4, 5). Life expectancy is variable and data in CdLS patients is limited. The largest study, reviewing deceased individuals from 1966–2007, reported mean ages at death of 12 years and 9 months for those surviving the neonatal period, 16 years and 2 months for those surviving beyond one year, and 28 years and 2 months for those surviving beyond 18 years; importantly, these findings reflect age at death rather than true life expectancy (6). The diagnosis of CdLS is based on the constellation of clinical features and/or molecular genetic testing. Sixty percent of CdLS patients have pathogenic variants in *nipped B-like* (*NIPBL*), which encodes the cohesin regulatory protein delangin and is associated with the severe “classic” phenotype, as in our patient (7). Variants in other cohesin-related genes, including *BRD4*, *SMC1A*, *SMC3*, *PDS5B*, *RAD21*, and *HDAC8* have also been identified as major contributors to CdLS (7). Over 20 additional causative genes have been reported, contributing to the wide array of clinical severity depending on the genotype (7).

Newborn screenings detect T-cell receptor excision circles (TRECs), DNA byproducts generated during T-cell receptor recombination in the thymus. Low or absent TREC levels indicate inability to produce or differentiate T-cells which is highly suggestive of Severe Combined Immunodeficiency (SCID) (8), the pathology that was initially suspected in this case. To our knowledge, this is the first detailed report of CdLS with severe transient T cell lymphopenia identified by newborn TREC screening.

## Case report

**Figure 1:**
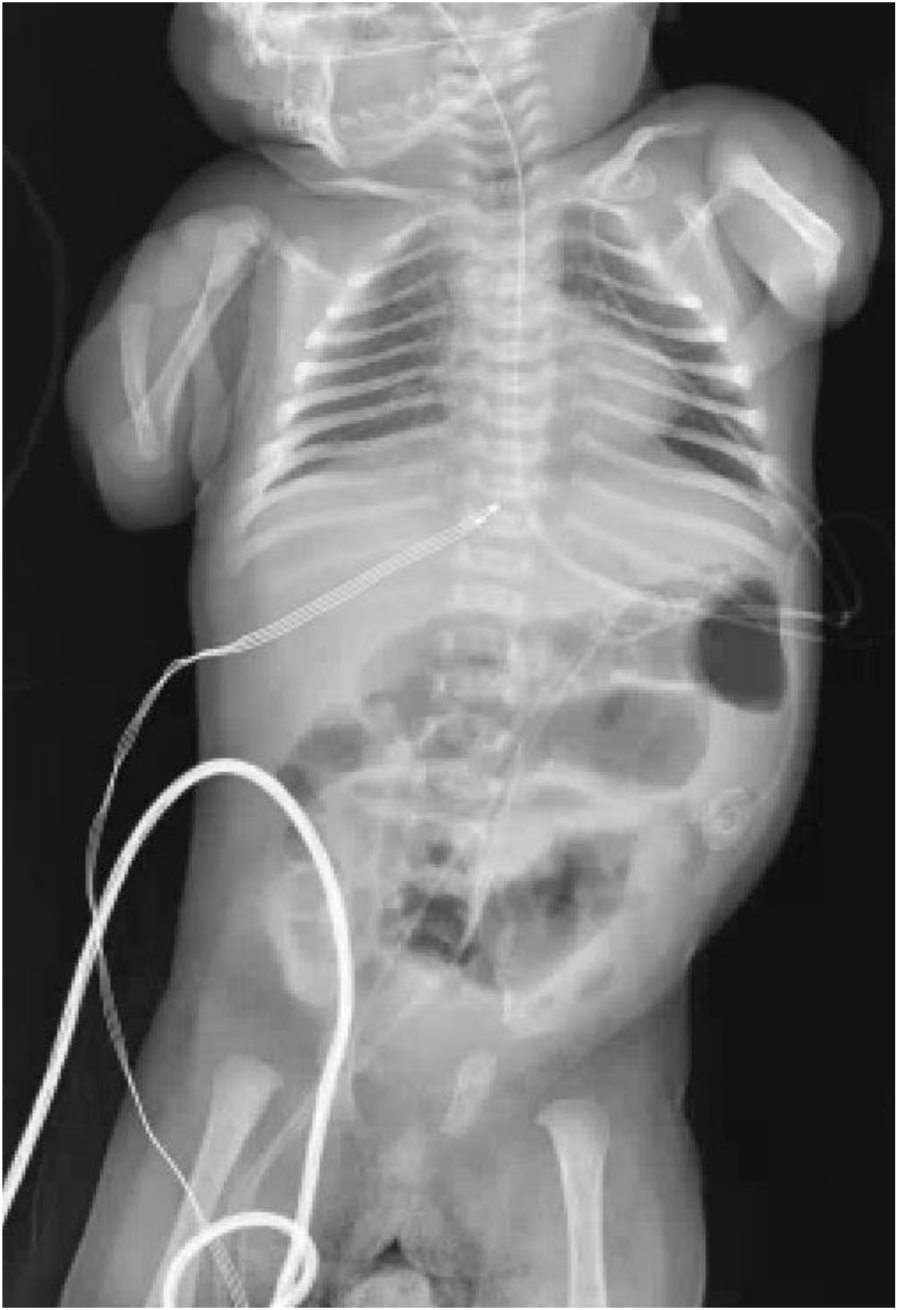
Bilateral upper extremity deformities with absent ulnae and dysplastic radii.

**Figure 2:**
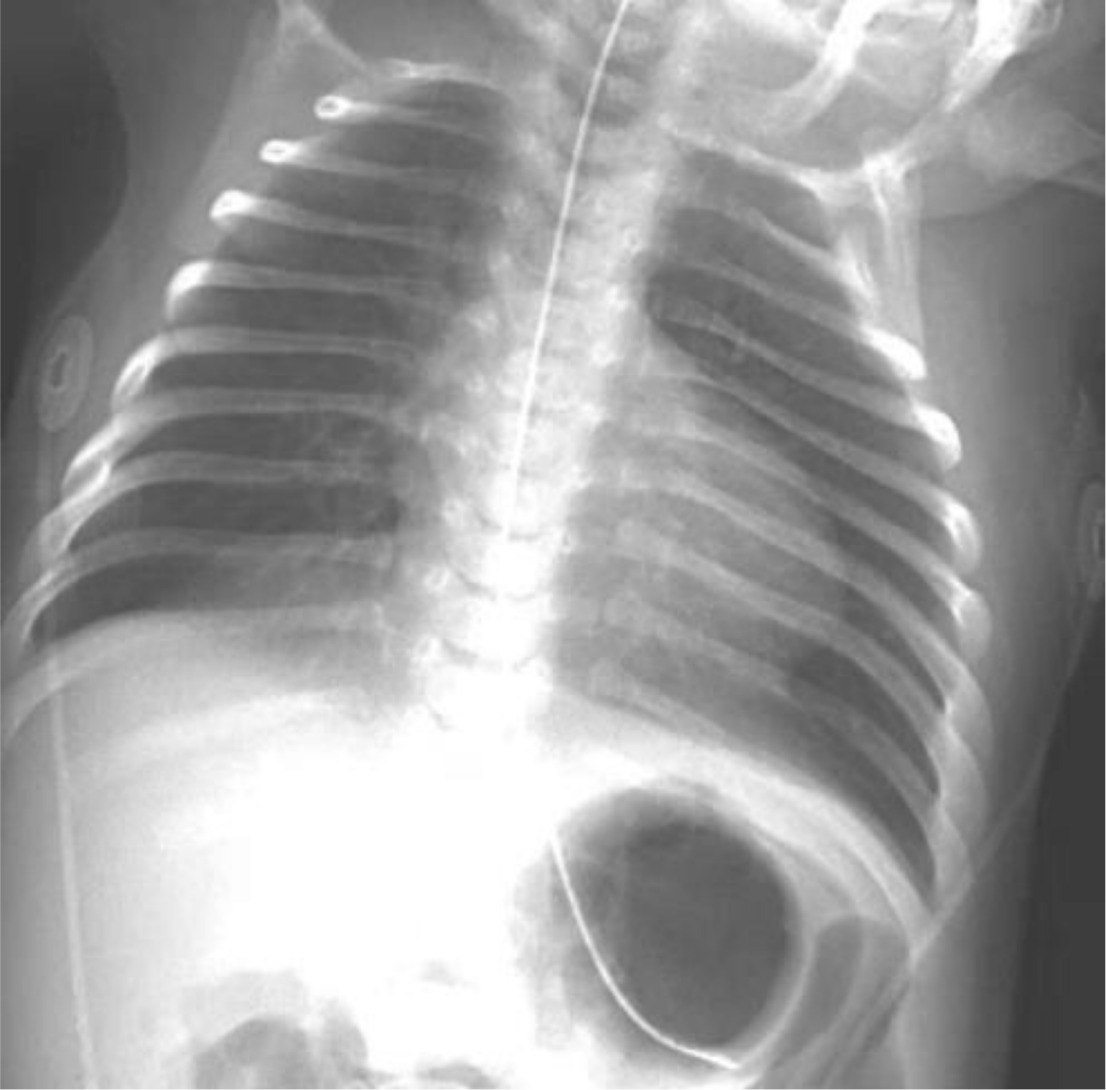
Frontal chest radiograph of an infant showing ribs, vertebral column, diaphragm, and part of the abdomen, with a nasogastric tube extending down the midline toward the stomach region. Thymic shadow is not evident.

A 2-week-old full-term female infant presented to our institution for further evaluation following an abnormal result on the newborn TREC screening, small-for-gestational-age status, and concern for cardiac, endocrine, genetic, and chromosomal abnormalities. Prenatal genetic evaluation demonstrated a female fetus with low-risk aneuploidy screening, including negative results for trisomy 21, 18, and 13, and normal maternal serum alpha-fetoprotein; additional screening (quad screen, cell-free DNA, and fetal fibronectin) was also low risk. Fetal ultrasonography revealed an abnormal calvarium, upper extremity hypoplasia, ventricular and atrial septal defects, pericardial effusion, and ventricular wall thickening. On exam, she exhibited a constellation of findings including severe limb reduction defects consisting of complete absence of the forearms, hirsutism, and dysmorphic facial features such as synophrys with arched and thick eyebrows, long and thick eyelashes, short and upturned nasal tip, anteverted nares, long and smooth philtrum, thin upper lip vermilion and downturned corner of the mouth. Skeletal survey demonstrated bilateral absence of the ulnae and shortened radii (**Figure 1**). Her exam was highly suggestive of classical CdLS with a clinical score of 15 points (5 cardinal and 5 suggestive features) (9). Chest X-ray showed paucity of thymic shadow (**Figure 2**). Cardiac anomalies included a patent foramen ovale and a single coronary origin of the left and right coronaries from the right coronary cusp. The patient’s initial immunologic workup demonstrated severe T-cell lymphopenia (CD3^+^ = 255 cells/µL) with low but detectable naïve CD4^+^ T cells (CD4^+^CD45RA^+^ = 115 cells/µL, 64%), low IgG (337 mg/dL) and IgA (<5 mg/dL), negative maternal engraftment studies, and normal lymphocyte proliferation in response to mitogens. She did not meet diagnostic criteria for SCID which is defined as having fewer than 50 autologous T cells/μL (10).

Given her clinical presentation, a CdLS gene panel including Next Generation Sequencing (NGS) and deletion/duplication analysis, was performed and revealed a variant of uncertain significance (VUS), c.6705_6707 deletion (p.Lys2235 del, NM_133433.3) in *NIPBL*. Sequencing was performed on genomic DNA using an Agilent targeted capture method, and amplified regions were sequenced with 2×150 bp reads on Illumina systems, with ≥20X coverage per base; exons were considered fully covered if all coding bases plus three nucleotides of flanking sequence were ≥20X. While this specific deletion has not previously been documented in a patient with CdLS, pathogenic variants within the corresponding region of *NIPBL* have been established as causative (7). Genome sequencing (GS) confirmed the same variant in *NIPBL*.

A primary immunodeficiency panel identified a heterozygous pathogenic *PAX1* variant, c.158C>A (p.Ser53*, NM_001257096.2), inherited from her mother. This panel used Illumina sequencing with >50X coverage, aligned to GRCh37, including coding regions plus 20 bp of flanking introns; exon deletions/duplications were called using an in-house read-depth algorithm. GS also identified a 1.4 Mb duplication at chromosome 10q21.3 (chr10:66,337,001–67,764,000) and a 1.2 Mb duplication at chromosome 7p21.2 (chr7:13,998,001–15,227,144), both classified as VUS. GS was conducted on a research basis at the HudsonAlpha Institute for Biotechnology (outside a CAP/CLIA environment) using an Illumina NovaSeq sequencer at an approximate depth of 30X. Variant pathogenicity was assessed according to ACMG guidelines. Maternal sample was provided only for comparison of the immunodeficiency gene panel, and no paternal samples were provided.

**Figure 3:**
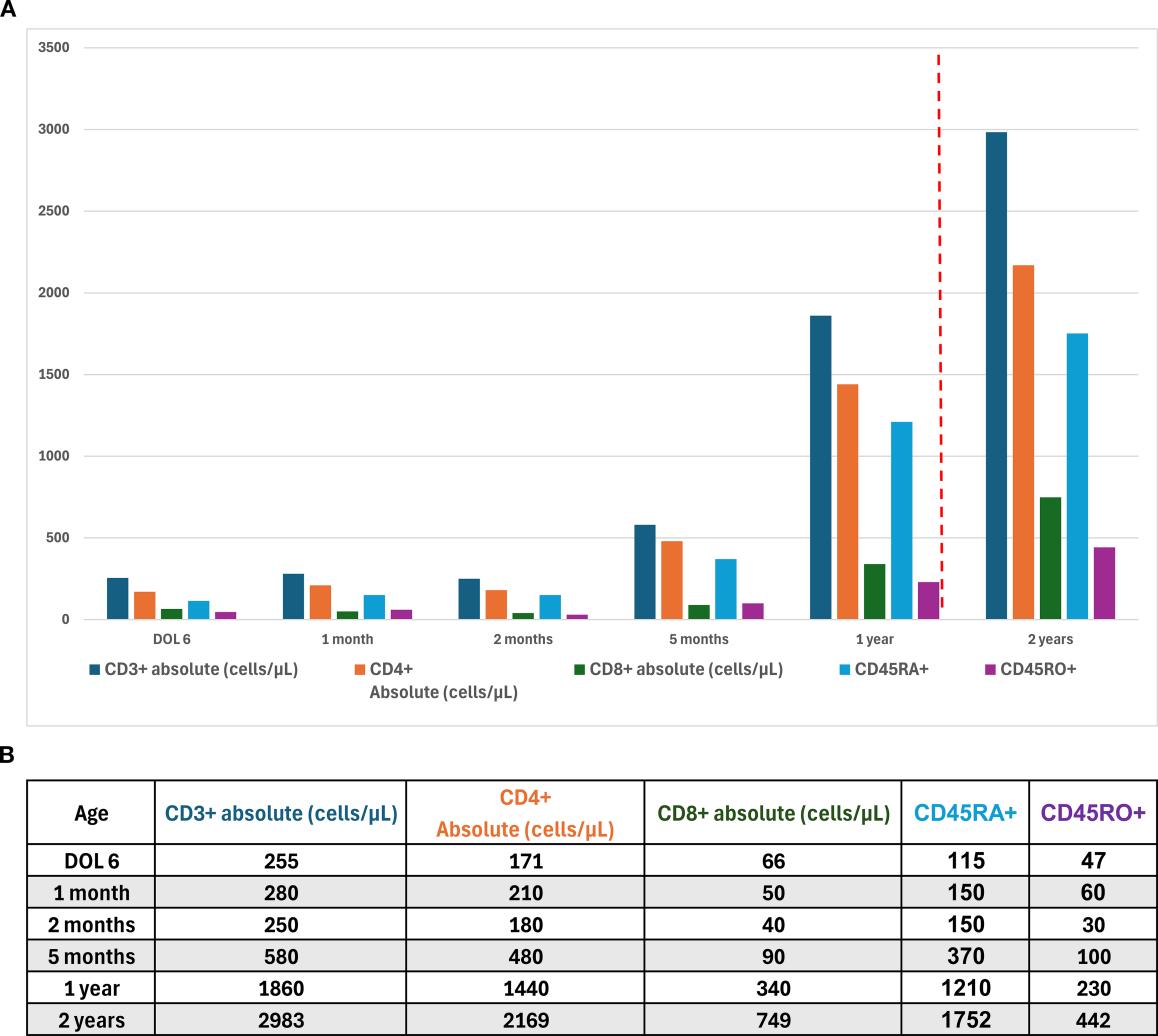
**(a)** T-cell counts measured over the first two years of life. The red dotted line indicates the time point at which antibacterial prophylaxis was discontinued (12 months of age). **(b)** T-cell counts measured over the first two years of life.

Due to the substantial immunologic findings, the patient was initiated on intravenous immunoglobulin (IVIG) and prophylactic antimicrobial therapy comprising sulfamethoxazole/trimethoprim, acyclovir, and fluconazole, the standard prophylaxis regimen used at our institution for patients with SCID or profound T cell defects. Vaccinations were deferred during IVIG, and household contacts received inactivated influenza vaccination but did not receive live vaccines. She was subsequently transitioned to subcutaneous immunoglobulin (SCIG) administered every other week, and antimicrobial prophylaxis was discontinued at approximately 1 year of age as T cell counts improved. Lymphocyte subpopulations were regularly monitored during this period (**Figures 3a**, **3b**). Following resolution of lymphopenia and the absence of severe infections during two years of treatment and follow-up, immunoglobulin therapy was discontinued. Immunoglobulin levels (IgG, IgA, IgM) were reassessed three months after discontinuation of SCIG and were found to be within normal limits. Post-therapy, the patient has experienced only occasional viral respiratory infections and has tolerated a special-needs daycare without any infection precautions in place. She continues to exhibit poor growth despite receiving full nutritional support by gastrostomy tube feeding, weighing only 6.6kg with a length of 67cm at age 4 years.

## Discussion

It has been demonstrated that patients with CdLS experience an increased frequency of infections including chronic otitis media, chronic viral respiratory infections, pneumonia, sinus infections, oral candidiasis, sepsis, and bacterial skin infections (1). A study evaluating 27 patients with severe CdLS found that 9 had antibody deficiency syndrome (1). Additionally, individuals with CdLS demonstrated significantly decreased percentages of regulatory T cells and helper T cells compared with healthy controls (P <.05) (1).

Several mechanisms likely contribute to the increased incidence of recurrent infections and detectable immune abnormalities in patients with CdLS. The cohesin protein complex is primarily known for its roles in sister chromatid cohesion and transcription regulation, but it also participates in DNA repair and gene interactions. Variable (V), diversity (D), and joining (J) gene recombination along with somatic hypermutation (SHM) are critical for generating diverse immunoglobulin variable (IgV) regions that enhance antibody-antigen affinity. B cells rely on receptor diversification to defend against a wide array of pathogens. DNA repair mechanisms play a crucial role in V(D)J recombination, SHM, and class-switch recombination (CSR). Data from one study suggest that cohesin-associated proteins NIPBL and Structural Maintenance of Chromosomes 1A (SMC1A) regulate V(D)J recombination in human B cells by modulating locus contraction or influencing DNA repair pathways (4). Abnormal CSR patterns have been observed in B cells from patients harboring *NIPBL* pathogenic variants (11). Therefore, reduced V(D)J recombination and abnormal CSR in CdLS patients with heterogeneous pathogenic *NIPBL* variants may contribute to their increased susceptibility to infections (4). The variant identified in our case, c.6705_6707del (p.Lys2235del), was classified as VUS. This alteration lies within the C-terminal HEAT-repeat domain, specifically in subdomain H4, a region critical for cohesin binding and function. Lys2235 is located within a highly conserved segment of the protein (approximately residues 1150–2650) across diverse vertebrate species, suggesting that this residue is evolutionarily important and likely contributes to the structural integrity and function of the HEAT-repeat domain (12). Mutations such as p.Lys2235del, occurring at conserved positions within key structural domains, are more likely to be pathogenic. Given its location in the HEAT repeats, such alterations may disrupt cohesin binding, chromatin association, or the conformational stability of *NIPBL*. Pathogenic variants affecting highly conserved residues in this region have been reported in patients with classical CdLS (13, 14). Additionally, an in-frame 6-bp deletion (c.6250_6255del, p.Val2084_Ser2085del), located within the same conserved region has also been reported in classical CdLS patients (14). Therefore, the in-frame deletion observed in our case could potentially disrupt cohesin binding and *NIPBL* function.

Additionally, expression of NFATc2 (nuclear factor of activated T cells, cytoplasmic, calcineurin-dependent 2), a key transcription factor involved in T-cell activation and T-cell receptor gene rearrangement, is decreased in CdLS patients (1). While profound T cell defects are not recognized as part of the syndrome, one publication mentions CdLS as an “extremely rare” syndrome to be captured by low TRECs without providing any additional details on the degree of insufficiency, medical management or patient outcome (15). In correspondence with the senior author on the aforementioned paper it was discovered that one previous case of CdLS with T cell lymphopenia was captured by TREC screening in the state of California, but no additional details are available. No such case reports could be found in the literature at the time of this manuscript.

A potentially confounding factor in this case is the identification of a pathogenic variant in *Paired Box Gene 1* (*PAX1)*. *PAX1* plays a critical role in the development and function of thymic epithelial cells, which are essential for normal T cell maturation. Biallelic loss-of-function mutations in *PAX1* have been associated with severe thymic hypoplasia or aplasia, leading to profound T cell immunodeficiency, as well as Otofaciocervical syndrome type 2—characterized by low-set cupped ears, preauricular fistulae, facial anomalies, shoulder girdle abnormalities, dental caries, skeletal anomalies, hearing loss, developmental delay and intellectual disability (16). This patient’s physical features were inconsistent with Otofaciocervical syndrome. Murine models harboring heterozygous pathogenic *PAX1* variants have not demonstrated thymic dysfunction or immunodeficiency. Furthermore, no reported cases in the literature link immunodeficiency or thymic hypoplasia to individuals with a single mutated *PAX1* allele (17). Maternal genomic testing revealed that the mother carries the same *PAX1* variant but has no anatomical abnormalities or history of recurrent infections. Considering the absence of clinical findings in the maternal carrier, it is unlikely that the heterozygous pathogenic *PAX1* variant is the cause of the immune abnormalities in this patient.

## Conclusion

Patients with CdLS have increased susceptibility to infections, likely due to pathogenic variants affecting genes that regulate the cohesin complex. While mild to moderate immune abnormalities have been documented in CdLS, to our knowledge, severe transient T cell lymphopenia identified through newborn TREC screening has not been previously published. This case underscores the utility of TREC screening in all infants, including those with underlying syndromes. Multi-institutional longitudinal studies are needed to better characterize the immune system in infants with CdLS and to determine whether improvement of the immune system with age is a common feature. Such studies are paramount in establishing immunologic treatment and monitoring recommendations for patients with CdLS.

## Data Availability

The original contributions presented in the study are included in the article/supplementary material. Further inquiries can be directed to the corresponding author.
